# The global variation of medical student engagement in teaching: Implications for medical electives

**DOI:** 10.1371/journal.pone.0229338

**Published:** 2020-02-24

**Authors:** Rhys D. Wenlock, Michael F. Bath, Tom Bashford, Katharina Kohler, Peter J. Hutchinson

**Affiliations:** 1 NIHR Global Health Research Group on Neurotrauma, Cambridge, England, United Kingdom; 2 School of Clinical Medicine, University of Cambridge, Cambridge, England, United Kingdom; 3 Centre for Neuroscience, Surgery and Trauma, Queen Mary University of London, London, England, United Kingdom; 4 Division of Anaesthesia, Cambridge Biomedical Campus, Addenbrooke's Hospital, NIHR Global Health Research Group for Neurotrauma, University of Cambridge, Cambridge, England, United Kingdom; 5 Division of Neurosurgery, Cambridge Biomedical Campus, Addenbrooke's Hospital, NIHR Global Health Research Group for Neurotrauma, University of Cambridge, Cambridge, England, United Kingdom; University of Newcastle, AUSTRALIA

## Abstract

**Introduction:**

International medical electives, whereby undergraduates visit an institution in a country other than their own, are a common part of medical training. Visiting students are often asked to provide local teaching, which may be acceptable where the visitor is acting within the bounds of their own competency and the normal practices of both their home and host institutions. However, the extent to which teaching is an accepted student activity globally has not previously been described. This study aims to address this using an international survey approach.

**Methods:**

A voluntary electronic survey, created using the Checklist for Reporting Results of Internet E-Surveys (CHERRIES) framework, was distributed across established international medical student networks. This assessed the involvement of medical students in teaching and the educator training they receive, with the intention of comparing experiences between high-income countries (HICs) and low/middle-income countries (LMICs) to gauge the engagement of both “host” and “visiting” students.

**Results:**

443 students from 61 countries completed the survey, with an equal proportion of respondents from LMICs (49.4%, 219/443) and HICs (50.6%, 224/443). Around two thirds of students reported providing teaching whilst at medical school, with most reporting teaching numerous times a year, mainly to more junior medical students. There was with no significant difference between LMICs and HICs. Around 30 per cent of all medical students reported having received no teacher training, including 40 per cent of those already providing teaching.

**Conclusion:**

This study suggests that students are engaged in teaching globally, with no difference between HIC and LMIC contexts. However, students are underprepared to act as educators in both settings. Providing teaching as part of an elective experience may be ethically acceptable to both host and home institutions, but needs to be supported by formal training in delivering teaching.

## Introduction

Teaching is central to the role of the modern-day clinician, as they share knowledge and experience with their fellow healthcare professionals, students, and patients. The General Medical Council (GMC) of the UK recognises this and requires the graduating medical student to be a “teacher for other learners in the multi-professional team” [1]. In high-income countries (HICs), medical students are often involved in the provision of teaching and with the increasing popularity of overseas medical electives, there are anecdotal reports of visiting students being asked to provide teaching while on their elective [2–4]. Whether such practice is ethical remains up for debate [5].

Up to now, much of the critical literature on medical electives focuses on the specific ethics of clinical practice with little emphasis on teaching. Whilst work has previously shown the benefits that medical student teaching can offer both teacher and student [6,7], the gaps in our knowledge surrounding current student teaching capacity limits our ability to understand the ethics of medical student teaching during electives. Understanding the extent to which medical students from across the world act as educators and whether they are qualified or experienced to do so is not only crucial in informing that debate but would also provide an insight into the state of global medical education. No previous work has sought to answer these questions.

To help address this, we conducted an international survey of medical students to address three key questions relating to their overall experience of teaching (1) Do medical students teach? (2) What is the pattern of medical student teaching? (3) Do medical students receive educator training? With that information, we set out to better understand the role of medical students in teaching provision on a global scale and, by comparing across High-Income (HIC) and Low-and-Middle Income (LMIC) countries, help inform the ethical debate of medical student teaching on electives.

This study will provide an insight into whether teaching falls within the responsibilities of medical students in both “home” and “host” settings (typically HICs and LMICs respectively) and therefore clarify, as per the UK’s Medical School Elective Alliance Council, the responsibilities of students in both their host and home nations.

## Methods

### Study design

This study was conducted using an open, voluntary, internet-based survey designed by the authors (RW/TB/MB) in line with the Checklist for Reporting Results of Internet E-Surveys (CHERRIES) framework [8]. A survey was created and disseminated through a structured electronic communication network involving electronic mailing lists and social media platforms of national and international student societies and medical schools. No participants were approached directly, nor were any incentives offered.

The questionnaire was hosted on an online survey platform (Qualtrics, Provo, United States), through an academic account held by the University of Cambridge, and ran from October 2018 to January 2019. Ethical approval for the study was granted by the Cambridge Psychology Research Ethics Committee (reference PRE.2018.070), with sponsorship provided by the University of Cambridge School of Clinical Medicine. The questionnaire was piloted prior to dissemination amongst medical students in the UK to assess functionality and usability.

All relevant information about the questionnaire and the study was provided to the participants before starting, and participants were able to withdraw from the analysis at any point. Any student reading medicine at a globally-recognised medical school, as classified by the World Directory of Medical Schools, was eligible to take part in this questionnaire, including graduate entry and intercalating students.

### Questionnaire design

A 23-item self-administered online questionnaire (text in S1 Survey) was created, sub-divided into four sections across 10 pages: (1) Consent; (2) Demographics; (3) Teaching Opportunities; (4) Providing & Receiving Peer-to-Peer Teaching. Questions required a combination of single response, multiple responses, and free text responses as appropriate.

Teaching roles were provided to participants in the questionnaire and defined consistently with previous published literature [9]:

- Peer to Peer Teaching: an individual (who is not a professional teacher) providing an educational lecture to students of similar social grouping to help each other learn.
- Mentoring Schemes: an individual (who is not a professional teacher) providing one-to-one teaching sessions to a select student of similar social grouping to help each them learn.
- Assisting Medical Faculty Teaching: an individual (who is not a professional teacher) providing assistance to medical faculty in a formally-organised teaching session for other students.

Questions required a variety of single response, multiple responses, and free text responses. Some questions had dependent responses, such as a free text options becoming available if the multiple-choice selection was “other (please specify)”.

### Data analysis

All responses were imported into the RStudio Statistical Package (v1.1.463, RStudio Inc., Delaware, USA) for analysis and visualisation. A descriptive analysis of completed responses was performed. The Internet Protocol (IP) addresses of respondents were recorded, with identical addresses being manually screened to check for duplication.

As the survey was created to explore the difference between HIC and LMIC respondents, a weighting was applied to account for variations in the response rate between different countries. Each country was given an equivalent weighting, with answers within each country averaged across all respondents (referred to as adjustment or adjusted analysis throughout the study).

Analyses were conducted first reflecting the respondents as a single global body of students, and secondly comparing responses between those studying in LMICs and HICs as defined by the World Bank [10]. Fisher’s exact test and the two-proportion Z-test were used to analyse the statistical significance between HICs and LMICs, for larger and smaller (less than 30 respondents per response category) subsets respectively.

## Results

### Responses

**Fig 1:**
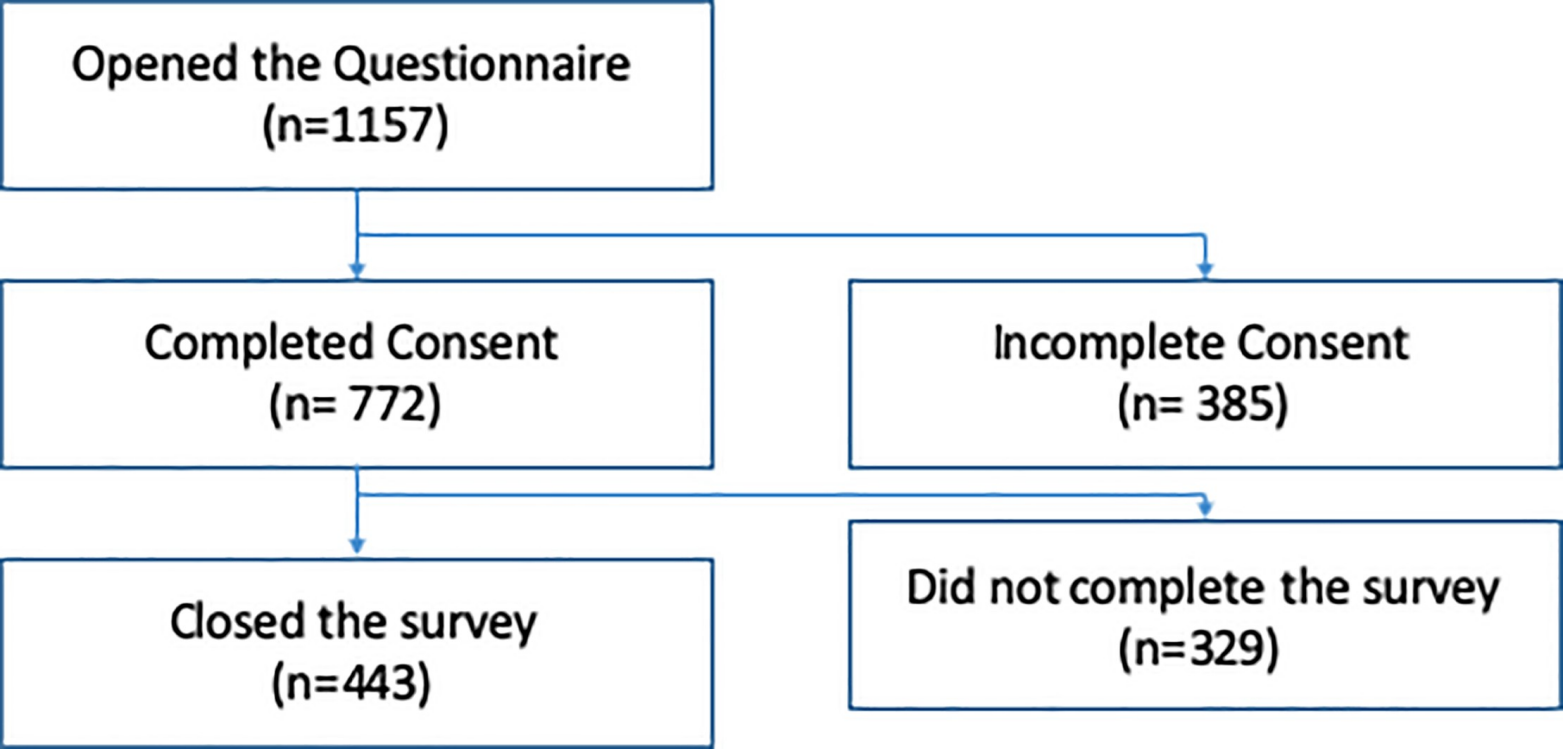
Flowchart outlining the questionnaire participation and completion rates.

The survey was opened initially by 1157 individuals, with 772 respondents (66.7%) providing their consent to participate. In total, 443 respondents (57.3%) completed the survey (Fig 1), including thirty-seven respondents who completed the questions but did not formally close the survey. Forty duplicate IP addresses were identified, all of which showed responses which varied sufficiently to be deemed unique and therefore included in the final analysis.

### Demographics

**Fig 2:**
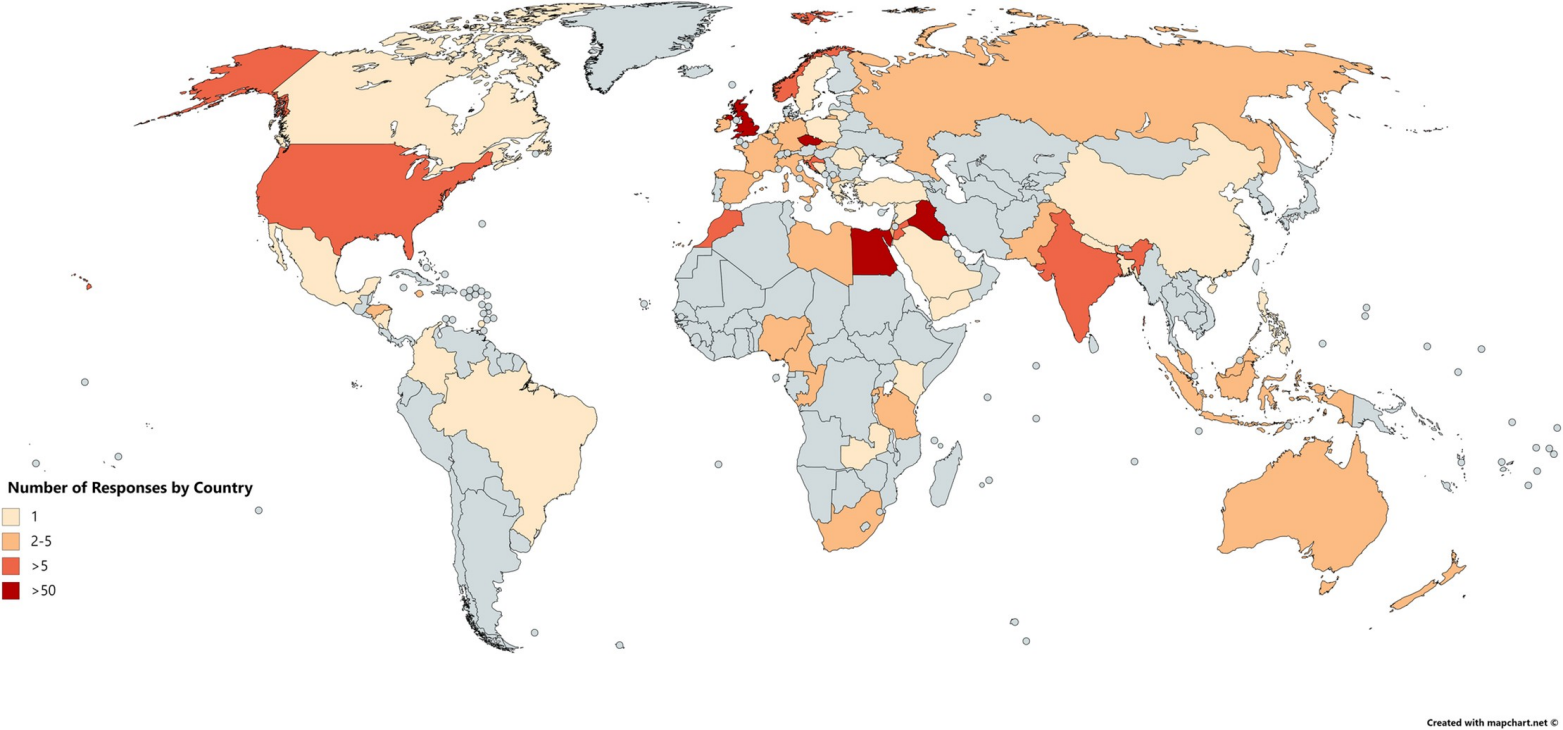
World map illustrating the distribution of responses by country (Reprinted from mapchart.net under a CC BY license, with permission from mapchart.net, original copyright 2019).

Sixty-one unique countries across all 6 habitable continents were represented in the questionnaire (Fig 2), with a median of 2 (IQR 1–4) responses per country; of note four countries (United Kingdom, Egypt, Czech Republic, and Iraq) provided 64.3% of total respondents. Overall, 224 students were affiliated with universities in High-Income Countries (HICs) and 219 students with universities in Low-and-Middle Income Countries (LMICs).

For 86% (381/443) of students, medicine was their first degree (Table 1). The median year of study for students responding to the survey was the 4th year and this was similar when comparing students from LMICs (IQR 2–5) and HICs (IQR 3–6). Those completing the survey from LMICs were more likely to be in their pre-clinical (37.6%, 82/219) stage of training, compared to their HIC counterparts (19.6%, 44/224, p <0.001). However, this effect was not present after adjustment (p = 0.22).

**Table 1:** The demographics of the 443 respondents, by year of study, degree entry and nature of study. *first degree in any subject. **after completing an initial degree. Teaching Provision.

Year of Study	LMIC	HIC	Total
*First (1st)*	11/219 (5.0%)	11/224 (4.9%)	22/443 (5.0%)
*Second (2nd)*	55/219 (25.1%)	29/224 (12.9%)	84/443 (19.0%)
*Third (3rd)*	43/219 (19.6%)	37/224 (16.5%)	80/443 (18.1%)
*Fourth (4th)*	46/219 (21.0%)	57/224 (25.4%)	103/443 (23.3%)
*Fifth (5th)*	28/219 (12.8%)	29/224 (12.9%)	57/443 (12.9%)
*Sixth (6th)*	17/219 (7.8%)	54/224 (24.1%)	71/443 (16.0%)
*Seventh (7th)*	10/219 (4.6%)	6/224 (2.7%)	16/443 (3.6%)
*Eighth (8th)*	5/219 (2.3%)	0/224 (0%)	5/443 (1.1%)
*Ninth (9th)*	4/219 (1.8%)	1/224 (0.4%)	5/443 (1.1%)
***Degree Entry***			
Undergraduate entry*	197/219 (90.0%)	184/224 (82.1%)	381/443 (86.0%)
Post-graduate**	22/219 (10.0%)	40/224 (17.9%)	62/443 (14.0%)
***Nature of Study***			
*Pre-clinical*	83/219 (37.9%)	44/224 (19.6%)	127/443 (28.7%)
*Mixed preclinical and clinical*	47/219 (21.5%)	35/224 (15.6%)	82/443 (18.5%)
*Clinical*	89/219 (40.6%)	145/224 (64.7%)	234/443 (52.8%)

**Fig 3:**
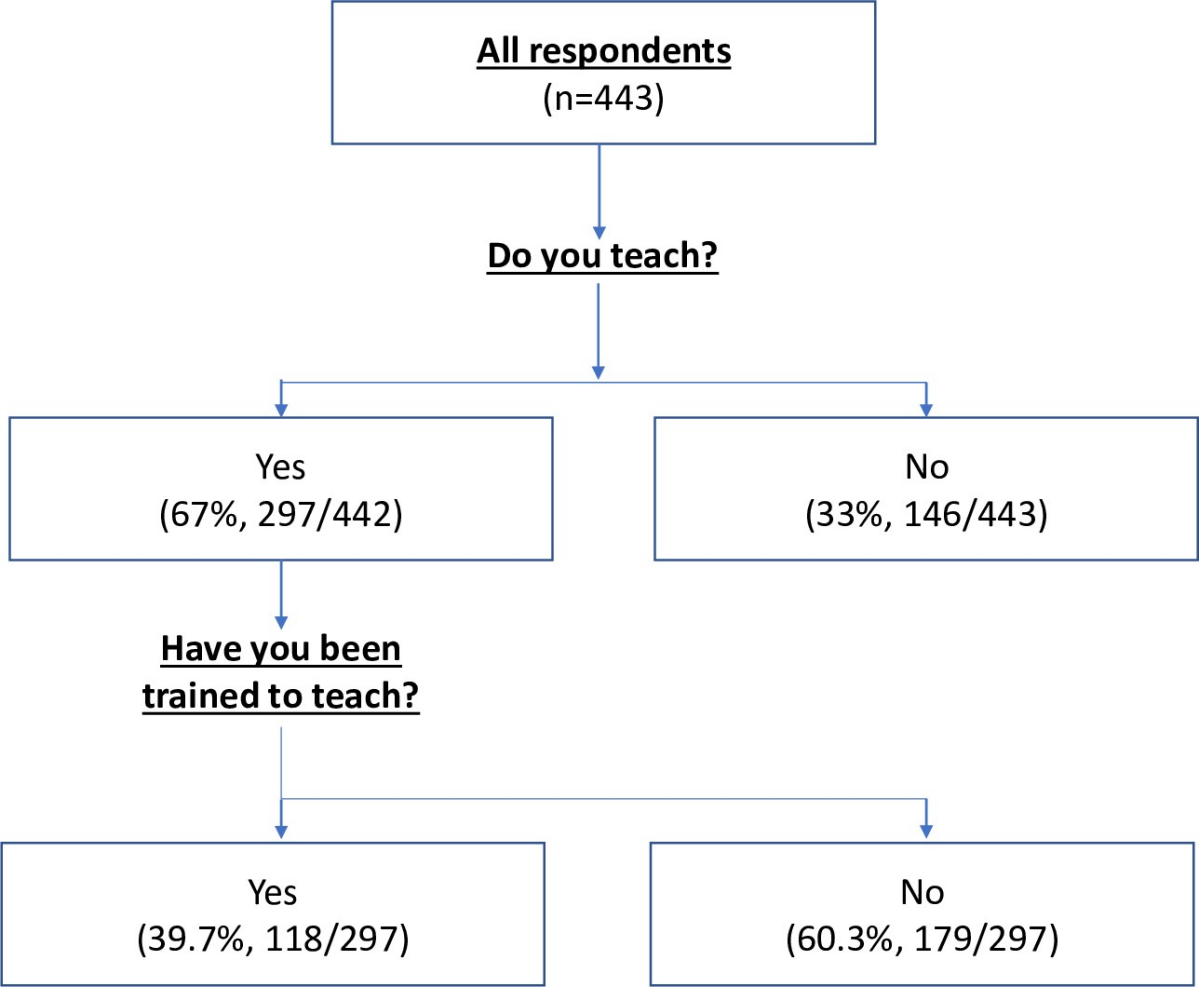
A flowchart outlining the number of students who reported teaching and whether they have received training.

Two thirds (67%, 297/443) of participants reported providing teaching during their medical training (Fig 3). When comparing the LMIC and HIC student cohorts, there was no statistically significant difference in the proportion of students acting as educators, with 62.6% (137/219) and 71.4% (160/224) respectively (p = 0.059). Following adjustment, similar proportions remained (71.4% versus 78.2%, p = 0.345). The earlier stage of training of respondents from LMICs did not completely explain the difference seen between the two contexts. When the LMIC year-group specific prevalence of teaching is applied to the year-group structure of the HIC cohort (therefore controlling for the varying distribution of year of study) the proportion of LMIC students who provided teaching was 65.4% compared to the initial value of 62.6%.

When exploring the type of teaching provided by students [9], peer-to-peer was the most common across both cohorts with 66.6% (198/297) providing this type of teaching. Mentoring schemes and assisting medical faculty teaching were less common with 38.7% (115/297) and 36.4% (108/297) respectively. The topic of teaching was most commonly “Clinical Knowledge” (72,4%, 215/297) followed by “Basic Science” (65.7%, 195/297), with a range of free text responses also being reported with examples including: “Surgical Skills”, “Anatomy”, “Practical Procedures,” and “Statistics”.

The frequency with which students engaged in teaching was similar between HICs and LMICs (Table 2). There was no significant difference in the proportion of students who taught “Regularly” or “Rarely” in either cohort (unadjusted p = 0.274, adjusted p = 0.183) (Table 2). The most common frequency of teaching was “Numerous times a year” in both cohorts (42.5% and 44.5% in HICs and LMICs respectively).

**Table 2:** The frequency of teaching provided by the 297 medical students that reported teaching. Rarely = “Never”, “Less than once a year”, and “Numerous times a year”. Regularly = “Around once a month”, “Around once a week”, and “Daily”.

Frequency of Medical Student Teaching	LMIC	HIC	p-value
***Rarely***	93/137 (67.9%)	98/160 (61.3%)	-
***Regularly***	44/137 (32.1%)	62/160 (38.7%)	0.183

The recipients of the medical student teaching were broadly similar between HICs and LMICs (Table 3). Students from LMICs were more likely to report teaching “Clinical Doctors” (24.8%, 34/137) than their HIC counterparts (15.0%, 24/160, p = 0.040) in the initial analysis, however following adjustment, this difference did not persist (p = 0.792). Similarly, HIC students were significantly more likely to report teaching “Medical Students (more junior than their level/grade/year)” than LMIC students (88.7%, 62.0%, p<0.001), however, again, this difference did not persist following adjustment (p = 0.971). After adjustment, LMIC students were however significantly more likely to report teaching “Other healthcare team members” (p = 0.007).

**Table 3:** The recipients of medical student-delivered teaching. *More junior than your level/grade/year. **More senior than/same level/grade/year as you.

Recipient of Teaching	HIC	LMIC	p-value
Medical Students*	142/160 (88.7%)	85/137 (62.0%)	0.971
Senior Medical Students**	68/160 (42.5%)	69/137 (50.4%)	0.069
*Clinical Doctors*	24/160 (15.0%)	34/137 (24.8%)	0.792
*Other healthcare team members*	17/160 (10.6%)	23/137 (16.8%)	0.007

### Reported benefits of teaching

**Fig 4:**
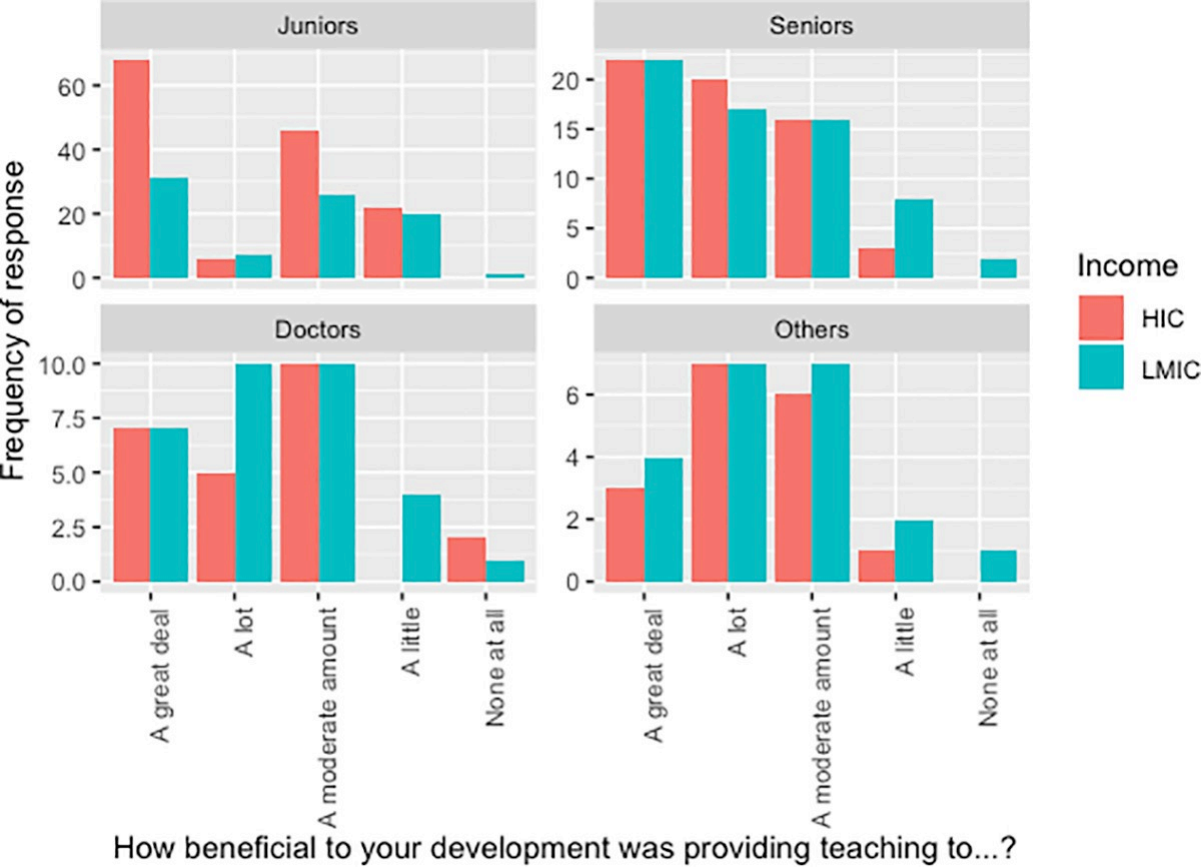
Graphic illustrating the perceived benefit of teaching different audiences.

Students were asked if they felt their teaching to be of benefit to their own development on a 5-point scale (Fig 4). Broadly, teaching any audience was felt to be beneficial, as illustrated by the low number of participants responding, “None at all”. However, teaching medical students may feel more beneficial, with the most common response for teaching Junior and Senior students alike being “1. A great deal”, whereas it was “3. A moderate amount” when the audience were Doctors. No significant differences were observed between respondents from HICs and LMICs.

### Training to teach

The majority of students did not receive any formal training to provide teaching (68.2%, 302/443). This persisted in those students who reported providing teaching (60.3%, 179/297, Fig 3). There was no significant difference in the prevalence of training between the HIC and LMIC students in the entire cohort (33.9% and 29.7%, p = 0.359 and adjusted p = 0.58), nor specifically in those who reported providing teaching (37.2% and 41.9%, p = 0.486 and adjusted p = 0.436). However, students that provided teaching were more than three times more likely to report having received training than their non-teaching counterparts (OR = 3.53, 95% CI 2.13–5.83).

Across respondents who received training, those studying in LMICs were statistically less likely to have been mandated to attend training by their medical school, with only 17% (11/65) reporting compulsory teaching, compared to the 38% (29/76) of HIC students (p = 0.008, adjusted p = 0.04). Similarly, of the students who had both received training and reported teaching, 18% (9/51) of students in LMICs were given compulsory training compared to 39% (26/67) in HICs (p = 0.015), showing an equally significant result after weighting (p = 0.003, Table 4).

**Table 4:** The type of training received by the 118 students who both reported teaching and received training.

What type of training was received?	LMIC	HIC	p-value
*Non-compulsory*	42/51 (82.4%)	41/67 (61.2%)	-
*Compulsory*	9/51 (17.6%)	26/67 (38.8%)	0.003

**Fig 5:**
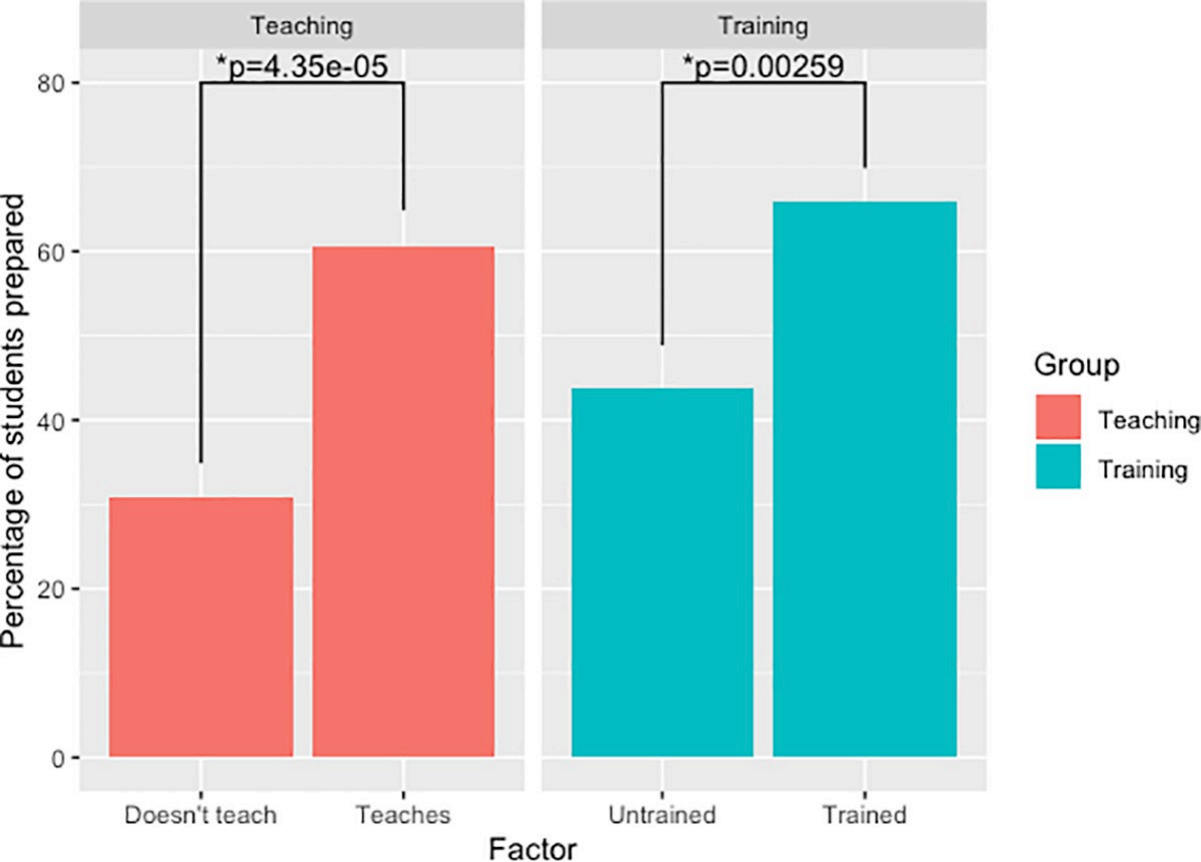
A graph showing how the proportion of students who feel prepared to teach is associated with A) teaching previously B) receiving training.

Students were asked if they felt prepared to act as a teacher or mentor; 223/443 (50.8%) reported feeling prepared to function in this way (Responses 1–3). This was significantly associated with having previously provided teaching (OR = 3.45, 95% CI 2.27–5.26) and having received any form of training previously (OR = 2.5, 95% CI 1.65–3.78) (Fig 5), both factors remaining significant after logistic regression (Previous Teaching OR = 3.00, 95% CI 1.95–4.62; Received Training OR = 1.99, 95% CI 1.29–3.07).

## Discussion

The word “doctor” originates from the Latin “docēre” meaning “to teach”, and this derivation still remains central to the role of the modern clinical doctor. In endeavouring to inform the ethical debate of teaching on elective, this study is the first to attempt to describe the pattern of medical student involvement in teaching on a global scale. Our study reports that medical students across the world are substantially involved in the provision of teaching, although many are not receiving any formal training in preparation. Furthermore, we found no differences in the rate of teaching provision, the pattern of teaching provided, or their preparation to act as educator exist between students in a LMIC compared to a HIC. While the medical elective raises complex ethical questions regarding the clinical practice of visiting students, these results suggest that engaging in teaching activities need not be seen as necessarily antithetical to local practice.

### Medical students as teachers

The published literature has repeatedly reported the benefits of peer-to-peer medical student teaching to the provider, including improved confidence and leadership abilities, greater knowledge and capacity to learn, and increased preparedness for their role in higher training [6,7]. Medical student-led teaching, whether faculty organised or not, has also been shown to be commonplace in High Income Countries [11] and reported results are often equivalent to faculty-led teaching [9].

Our data shows that the conception of students as educators is consistent between both HIC and LMIC contexts, and that the preparation and expectation of students to teach others is broadly similar at a global level. Similar to previously reported rates in HICs [11], our results suggest that around two thirds of medical students globally are engaged in teaching, with no significant difference between HICs and LMICs.

This raises immediate problems, with existing work demonstrating that some medical students show a lack of preparation to fulfil the role of educator [12,13]. Our work concurs with the previously published literature, as globally only 3 out of 10 medical students had received any form of educator training, increasing to 4 out of 10 for those who reported to have previously provided teaching in any form. When training was provided, it was statistically more likely to have been compulsory in HICs than LMICs.

If medical schools are to fulfil their function in equipping students to be the educators of the future, there would appear to be work to be done in understanding the barriers to effective teacher training and overcoming them. To do so, it is likely that medical institutions will have to develop and implement evidence-based teacher training programmes to support the already prevalent teaching provided by medical students.

### Implications for the medical elective

During medical electives, visiting students have previously been encouraged to provide local teaching [14]. This can be for a number of reasons, not least because they are seen as providing a window into the world of resource-intensive medical care and a witness to a perceived international standard [15]. However, this carries with it complex ethical challenges, involving the hosts’ perception of the visitor’s insight and experience, their ability as an educator, and the appropriateness of their teaching to the host context.

Although the data presented in this study focusses on the overall experience of medical student teaching it provides evidence to inform the ethical discussion of elective teaching. Particularly as the UK’s Medical School Elective Alliance Council explicitly recommends that students from HICs should act within the responsibilities of a student in both the host and their home nations when on elective [2]. Our study helps to provide an insight into whether teaching falls within the responsibilities of medical students in both “home” and “host” settings. Crucially, our work supports the idea that medical students are seen as educators in a variety of LMIC and HIC settings, and that they are similar prepared (or underprepared) to do so.

This raises the possibility of the medical elective being used as not only a chance to observe clinical practice in an unfamiliar setting, but also an opportunity to develop proven skills as an educator while also supporting the local healthcare system [16]. Of course, this will vary on a case-by-case basis and depend on accepted practices in both the host and visitor home contexts [17]. Therefore, there remains a crucial role for both home and host medical schools to support students in participating in their elective, particularly if teaching is to be a fundamental aspect of the elective experience.

Crucially, electives should not be seen as a mechanism for students from HICs to parasitise the clinical and cultural richness of LMIC settings. Rather, they should be bidirectional exchanges whereby students from any culture can visit a different one in order to challenge their own clinical, and educational, skills and knowledge [4,18]. This has the opportunity for mutual gain, but also requires mutual investment.

### Future work

This study represents the first attempt to describe a global pattern of teaching practice by medical students and further a comparison between high and low-income environments. We have attempted to apply rigour to a notoriously biased mechanism of data collection, however despite this, several limitations were present that should be acknowledged. Despite distribution by international networks, responses were not equally distributed across countries. We attempted to account for this using a weight-adjusted analysis in our results. To avoid issues with comparing translated versions of complex questions, the survey was only prepared in English, limiting its global reach. Furthermore, there is the potential for sampling bias within our analysis (e.g. it is possible that students who provide teaching were more likely to respond to our questionnaire). Despite this our results are similar to that of the previously published literature and some parts of the questionnaire were less likely to introduce sampling bias (e.g. the receipt of training previously) and therefore may more accurately reflect the characteristics of the true population [11]. As with any survey study design, our results are limited by recall bias.

Importantly however, this study provides significant information on the global levels of medical student involvement in teaching and supports the notion that teaching is an ethical component of medical electives, when local practices and individual experience are factored in. However, today’s medical students globally receive little training on how to be effective educators, with little variation between HICs and LMICs. This needs to be addressed if they are to deliver on the expectations placed upon them as teachers.

## Supporting information

S1 Survey The survey distributed in this analysis. (DOCX)

S1 Data Underlying data for this analysis and conclusions. (CSV)
