# Supplementary material for: The global variation of medical student engagement in teaching: Implications for medical electives
Source: PLoS One. 2020 Feb 24;15(2):e0229338. doi: 10.1371/journal.pone.0229338 (PMC7039511; doi:10.1371/journal.pone.0229338)
Supplement: S1 Survey — (DOCX) [file pone.0229338.s001.docx]

Medical Student Teaching Survey

Start of Block: Block 3

This survey is part of a research project run by the NIHR Global Health Research Group on Neurotrauma, a multinational team researching the improvement of neurotrauma care in low and middle-income countries.    As a group, our mission is to improve global neurotrauma care. We aim to achieve this through a series of projects and activities focussing on various aspects of traumatic brain injury (TBI) care in low and middle income countries (LMICs). More information can be found at www.neurotrauma.world. 

 We are interested in the role that medical students can play in both clinical improvement and academic research. Teaching is an essential element of these, and we want to better understand the experience medical students across the world get in teaching others.

 Thank you.
 NIHR Global Health Research Group on Neurotrauma     *Principle Investigator - Professor Peter Hutchinson, NIHR Research Professor at the Department of Clinical Neuroscience, University of Cambridge*


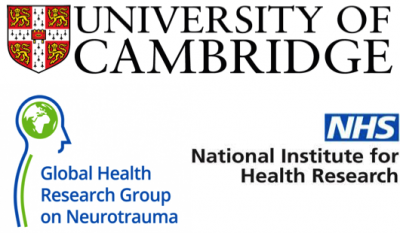


End of Block: Block 3

Start of Block: Block 5

This survey aims to investigate the role medical students currently have in medical care and the experiences of teaching they have had as part of their medical training. We are keen to understand what medical students current role and involvement in teaching and medical education is, and what further potentials there could be.   Completing this survey is entirely voluntary. The survey can be completed at any time between October-November 2018, but can be only completed once per participant. You can withdraw at any time, with no penalty or loss, now or in the future.   It is entirely anonymous and data will be stored securely for 5 years before being destroyed, with no individual data published. We hope to publish the findings internationally from this survey following its completion. If you wish to withdraw from the study following completion (prior to the end of the study period), please email info@neurotrauma.world   The study is being co-ordinated by the NIHR Global Health Research Group on Neurotrauma, with support from student groups Incision and Students for Global Health. The project has received ethical approval from the Psychology Research Ethics Committee of the University of Cambridge.
 If you require any further information, please go to www.neurotrauma.world or contact us via info@neurotrauma.world   By clicking next, you are stating you have read and understood the above information, are a current medical student, and are happy to continue with the survey.

End of Block: Block 5

Start of Block: Demographics

| 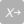 |
| --- |

Q1 In what country are you currently studying?

▼ Afghanistan (1) ... Zimbabwe (1357)

Q2 What was your course entry?

- Post-graduate entry (i.e. after completing an initial degree) (1)
- Undergraduate entry (i.e. first degree in any subject) (2)

Q3 What is current nature of your medical studies?

- Pre-clinical (1)
- Clinical (2)
- Mixed preclinical and clinical (3)

Q4 What is your current year of medical study (including intercalated degrees)?

- First (1st) (1)
- Second (2nd) (2)
- Third (3rd) (3)
- Fourth (4th) (4)
- Fifth (5th) (5)
- Sixth (6th) (6)
- Seventh (7th) (7)
- Eighth (8th) (8)
- Ninth (9th) (9)

End of Block: Demographics

Start of Block: Teaching Opportunities

Q5 Whilst at medical school, what formal teaching activities have you been actively involved in **providing**? Choose all that apply
*Definitions (*adapted from Yu et al., PMID: 23745087)*:*An individual (who is not a professional teacher) providing an educational lecture to students of similar social grouping to help each other learn **An individual (who is not a professional teacher) providing one-to-one teaching sessions to a select student of similar social grouping to help each them learn ***An individual (who is not a professional teacher) providing assistance to medical faculty in a formally-organised teaching session for other students*

- Peer-to-peer lectures* (1)
- Mentoring schemes** (2)
- Assisting medical faculty teaching*** (3)
- Other (please specify) (4) ________________________________________________
- Have not provided any teaching (5)

Q6 Are you encouraged to deliver teaching or mentoring by your institution?

- Yes (1)
- No (2)

Q7 What best describes how often you are **required** to deliver teaching or mentoring by your medical school?

- Never (1)
- Less than once a year (2)
- Numerous times a year (3)
- Around once a month (4)
- Around once a week (5)
- Daily (6)

Q8 Have you ever received formal training in teaching skills whilst at medical school? Choose all that apply

- No (1)
- Yes, non-compulsory & organised by an external group (2)
- Yes, non-compulsory & organised by the medical school (3)
- Yes, compulsory & organised by the medical school (4)

Q9 Do you feel you are adequately prepared to be able to function effectively as a teacher or mentor?

- A great deal - "I am definitely prepared" (1)
- A lot - "I am well prepared" (2)
- A moderate amount - "My preparation is adequate" (3)
- A little - "I need more preparation" (4)
- None at all - "I feel underprepared" (5)

End of Block: Teaching Opportunities

Start of Block: Providing Peer-To-Peer Teaching

Q10 Whilst at medical school, who have you **received** teaching sessions from? Choose all that apply

- Medical students, more junior than your level/grade/year (1)
- Medical students, more senior than (or same as) your level/grade/year (2)
- Doctor (3)
- Other members of the healthcare team (4)
- Other (please specify) (5) ________________________________________________
- I have not received any teaching (6)

Skip To: Q12 If Whilst at medical school, who have you received teaching sessions from? Choose all that apply = I have not received any teaching

Q11 How beneficial to your own clinical development did you find **receiving** teaching sessions from junior medical students?

- A great deal (1)
- A lot (2)
- A moderate amount (3)
- A little (4)
- None at all (5)
- Not Applicable (6)

Q12 How beneficial to your own clinical development did you find **receiving** teaching sessions from senior or same level medical students?

- A great deal (1)
- A lot (2)
- A moderate amount (3)
- A little (4)
- None at all (5)
- Not Applicable (6)

Q13 How beneficial to your own clinical development did you find **receiving** teaching sessions from doctors?

- A great deal (1)
- A lot (2)
- A moderate amount (3)
- A little (4)
- None at all (5)
- Not Applicable (6)

Q14 How beneficial to your own clinical development did you find **receiving** teaching sessions from other members of the healthcare team (e.g. non-doctors/medical students)?

- A great deal (1)
- A lot (2)
- A moderate amount (3)
- A little (4)
- None at all (5)
- Not Applicable (6)

| Page Break |  |
| --- | --- |

Q15 Whilst at medical school, who have you **provided** teaching sessions to? Choose all that apply

- Medical students, more junior than your level/grade/year (1)
- Medical students, more senior than (or same as) your level/grade/year (2)
- Clinical doctors (3)
- Other members of the healthcare team (4)
- Other (please specify) (5) ________________________________________________
- I have not provided any teaching (6)

Skip To: End of Block If Whilst at medical school, who have you provided teaching sessions to? Choose all that apply = I have not provided any teaching

Q16 How beneficial to your own clinical development did you find **providing** teaching sessions to junior medical students?

- A great deal (1)
- A lot (2)
- A moderate amount (3)
- A little (4)
- None at all (5)
- Not Applicable (6)

Q17 How beneficial to your own clinical development did you find **providing** teaching sessions to senior medical students?

- A great deal (1)
- A lot (2)
- A moderate amount (3)
- A little (4)
- None at all (5)
- Not Applicable (6)

Q18 How beneficial to your own clinical development did you find **providing** teaching sessions to doctors?

- A great deal (1)
- A lot (2)
- A moderate amount (3)
- A little (4)
- None at all (5)
- Not Applicable (6)

Q19 How beneficial to your own clinical development did you find **providing** teaching sessions to other members of the Healthcare team (e.g. non-doctors/medical students)?

- A great deal (1)
- A lot (2)
- A moderate amount (3)
- A little (4)
- None at all (5)
- Not Applicable (6)

| Page Break |  |
| --- | --- |

Q20 If you do provide teaching as a student, what best describes how often you teach?

- Never (1)
- Less than once a year (2)
- Numerous times a year (3)
- Around once a month (4)
- Around once a week (5)
- Daily (6)

Q21 If you do engage in teaching as a student, what kind of teaching have you **provided**? Choose all that apply

- Basic science (1)
- Clinical knowledge (2)
- Other (please specify) (3) ________________________________________________

Q22 If you do engage in teaching, do you (choose all that apply):

- Design and deliver the teaching sessions and content yourself (1)
- Deliver prescribed content in your own chosen style (2)
- Deliver prescribed content in a prescribed style (3)

Q23 How do you ensure quality control of your teaching? 
For example, formal or informal feedback, review by a senior clinician, mapping content delivered against the curriculum, or assessing students against knowledge or competency criteria

________________________________________________________________

End of Block: Providing Peer-To-Peer Teaching

Start of Block: Block 4

Q24 Thank you for completing this survey. If you wish to discuss any aspects of this study, please email us at info@neurotrauma.world or rdw39@cam.ac.uk

End of Block: Block 4
